# Oropharyngeal administration of colostrum targeting gut microbiota and metabolites in very preterm infants: protocol for a multicenter randomized controlled trial

**DOI:** 10.1186/s12887-023-04346-x

**Published:** 2023-10-16

**Authors:** Na Wang, Jia Zhang, Zhangbin Yu, Xudong Yan, Lian Zhang, Haibo Peng, Cheng Chen, Rui Li

**Affiliations:** 1 Department of Neonatology, The Affiliated Suqian First People’s Hospital of Nanjing Medical University, Jiangsu, China; 2 Department of Neonatology, Shenzhen People’s Hospital (The Second Clinical Medical College, Jinan University, The First Affiliated Hospital, Southern University of Science and Technology), Shenzhen, Guangdong China; 3 Department of Neonatology, Bao’an Maternal and Child Health Hospital, Shenzhen, Guangdong China; 4 Department of Neonatology, Longgang Maternal and Child Health Hospital, Shenzhen, Guangdong China

**Keywords:** Preterm newborn, Colostrum, Oral care, Microbiota, Metabolome

## Abstract

**Background:**

Oropharyngeal administration of colostrum (OAC) has an immune-stimulating effect on oropharyngeal-associated lymphoid tissue, and can promote the maturation of the gastrointestinal tract. However, how OAC promotes intestinal maturation in preterm infants by altering gut microbiota remains unclear. We aim to assess changes in gut microbiota and metabolites after OAC in very preterm infants.

**Methods:**

A multicenter, double-blind, randomized controlled trial will be conducted in three large neonatal intensive care units in Shenzhen, China, with preterm infants with gestational age less than 32 weeks at birth and birth weight less than 1500 g. It is estimated that 320 preterm infants will be enrolled in this study within one year. The intervention group will receive oropharyngeal administration of 0.2 ml colostrum every 3 h, starting between the first 48 to 72 h and continued for 5 consecutive days. Following a similar administration scheme, the control group will receive oropharyngeal administration of sterile water. Stool samples will be collected at the first defecation, as well as on the 7th, 14th, 21st and 28th days after birth for analysis of effect of OAC on gut microbiota and metabolites through 16sRNA gene sequencing and liquid chromatography-mass spectrometry.

**Discussion:**

This proposal advocates for the promotion of OAC as a safe and relatively beneficial protocol in neonatal intensive care units, which may contribute to the establishment of a dominant intestinal flora. Findings of this study may help improve the health outcomes of preterm infants by establishment of targeted gut microbiota in future studies.

**Trial registration:**

NCT05481866 (registered July 30, 2022 on ClinicalTrials.gov).

## Background

Human milk can improve the health outcomes of preterm infants and reduce the risk of necrotizing enterocolitis (NEC) and late onset sepsis (LOS) in early life [1]. This is because of bioactive components tin beneficial breast milk [2], especially colostrum, which expresses essential biological factors such as fatty acids, hormones, immunoglobulin, lactoferrin and oligosaccharides [3–5]. These beneficial biological factors in colostrum also seem to have a unique preventive effect on allergic diseases and chronic diseases later life [6, 7].

Early colostrum feeding can have many benefits for preterm infants. However, some very low birth weight infants often exhibit unstable clinical symptoms such as unstable breathing, poor sucking and poor gastrointestinal function shortly after birth. Therefore, fasting or the slow progress of enteral feeding is a common clinical manifestation of preterm infants [8]. Thus, these preterm infants do not receive all protective biological factors provided by human milk in the early stage of life. In addition, even though some preterm infants can be fed with human milk at an early stage, they need nasal feeding instead of direct oral feeding because of uncoordinated sucking and swallowing function [9]. This prevents establishment of protective biological factors in the oropharynx of very preterm infants within the first few weeks of life, predisposing them to many diseases in early life [10].

Oropharyngeal administration of colostrum (OAC) can be used as a natural alternative administration technique. In OAC, a small amount of colostrum rich in biological factors is injected into the surface of oral mucosa to stimulate an immunostimulatory effect on the infant's oropharyngeal-associated lymphoid tissue [11, 12], including promoting the absorption of secretory immunoglobulin A and lactoferrin [13]. In addition, it can promote gastrointestinal immune function and systemic anti-infection ability, thereby promoting the maturation of the gastrointestinal tract [14, 15].

An optimized human gut microbiota composition is one of the signs of optimal maturation of the gastrointestinal tract and plays a key role in regulating the health and disease risk in preterm infants [16, 17]. With recent advances in high-throughput multiomics technology, including metagenomics and metabonomics, as well as the measurement of host diseases and microbiota, many bacteria and bacterial products causing human diseases (such as inflammatory bowel disease and type 2 diabetes) have been identified [18]. Many intestinal microorganisms and metabolites are involved in the pathogenesis of LOS and NEC in preterm infants [19, 20]. Although LOS pathogens are diverse (including Staphylococci and Enterobacteriaceae and obligate anaerobes), they are typically enriched a few days before the onset of the disease and produce unique metabolites (such as ethanol and formic acid) [21, 22].

Before diagnosis of NEC, notable differences in enrichment of gut microbiota occur. The gut becomes enriched with Proteobacteria dominated by Enterobacteriaceae, whereas Firmicutes, dominated by Staphylococcus, is relatively lacking. As a result, there is excessive growth of pathogenic bacteria under specific conditions and the reduction of biodiversity. Microbiome optimization may provide a new strategy for the prevention of NEC [23, 24]. In addition, studies have explored preclinical diagnostic value of fecal metabolites (such as short chain fatty acids, including acetate and butyrate) for NEC [25, 26].

Although evidence is inadequate and the mechanism is not clear, gut microbiota and metabolites are potentially useful as biomarkers for early diagnosis of NEC and LOS [27]. Therefore, it is beneficial to identify customized mode of intestinal microorganisms and the biological mechanism underlying metabolite changes before NEC and LOS onset [28, 29].

The microbiome-metabolome association is bidirectional [30]. Host and gut microbiota interact through either symbiosis or imbalance of small molecule metabolites. Metabonomics is undoubtedly a promising method to study the interaction between host and gut microbiota [31]. Metabolites of bacteria may also be novel therapeutic targets for human chronic diseases [32]. Several previous studies have investigated the effect of OAC on enteral feeding, NEC, LOS, mortality, etc. [33–39]. Because of limited sample size and inconsistent OAC measures, among other reasons, there is no sufficient evidence to prove that OAC can reduce the incidence of NEC, LOS and death [40]. But OAC can promote intestinal maturation and rapidly achieve total enteral feeding [41, 42]. However, the mechanism by which OAC promotes intestinal maturation by changing intestinal microbiota and metabolites remains elusive, which is the significant contribution of our study.

We hypothesized that OAC could promote relative enrichment of intestinal Firmicutes but reduce relative enrichment of Proteobacteria. In addition, the production of short chain fatty acids and other metabolites would change accordingly to resemble the gut microbiota profile in normal full-term breast fed infants, improving the health outcomes of preterm infants. The purpose of this multicenter, double-blind, randomized controlled trial is to evaluate whether OAC can modulate gut microbiota diversity and metabolites (such as short chain fatty acids) compared with oral care with sterile water.

## Methods

This study is based on the standard protocol items: recommendations for interactive trials (SPIRIT) [43].

### Study design

**Fig. 1:**
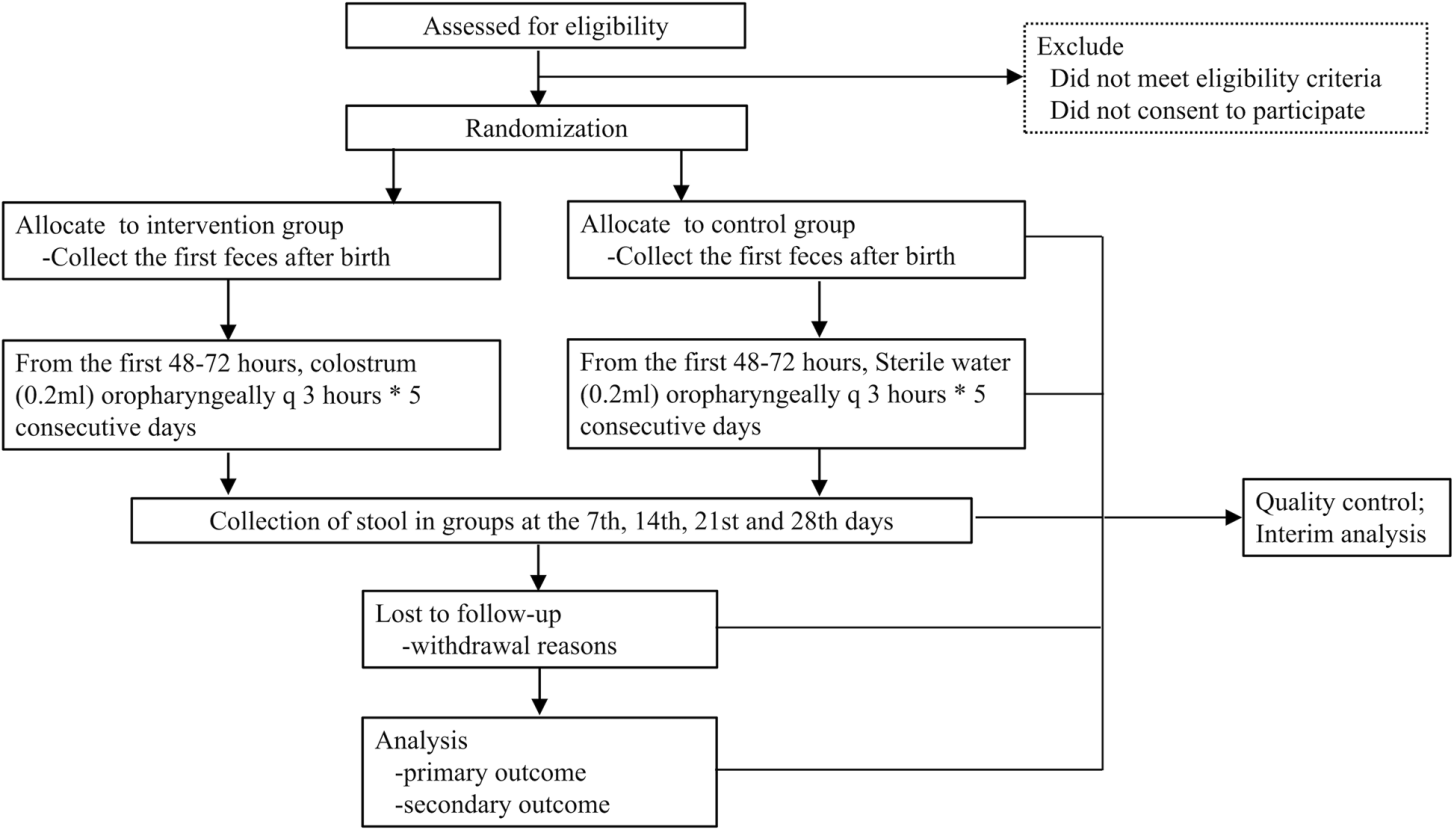
Flow diagram of the research process

This multicenter, double-blind, randomized controlled trial will be conducted in three large neonatal intensive care units (NICUs) in Shenzhen, Guangdong Province, China, from October 1, 2022 to September 31, 2023. The purpose of this study is to evaluate the effect of OAC on gut microbiota and metabolites. Preterm infants with gestational age < 32 weeks and weight < 1500 g will be evaluated. Infants who meet these inclusion criteria will be randomly divided into two groups in a ratio of 1:1. The intervention group and control group will receive 0.2 ml oropharyngeal colostrum and 0.2 ml sterile water, respectively, every three hours for five days. Stool samples will be collected at the first defecation, as well as on the 7th, 14th, 21st and 28th days to detect the gut microbiota and metabolites. A flow diagram of the research process including the planned study phases is shown in Fig. 1.

Subjects will be recruited from three level III NICUs in Shenzhen, Guangdong Province, China: 1) Shenzhen People's Hospital (about 6000 births in 2021 and a preterm birth rate of about 11%), 2) Longgang Maternal and Child Health Hospital (about 13,000 births in 2021 and a preterm birth rate of about 6.7%), and 3) Bao’an Maternal and Child Health Hospital (about 18,000 births in 2021 and preterm birth rate of about 7%). Shenzhen People’s Hospital designed and initiated the study. It is the coordinating center and the affiliation of the sponsor (ZY). These three NICUs are all located in economically developed areas in China, provide prenatal care, delivery, postnatal care, neonatal treatment and other services.

### Inclusion/exclusion criteria

Inclusion criteria: 1. gestational age less than 32 weeks and birth weight less than 1500 g; 2. admission to NICU ≤ 24 h; and 3. be able to start the agreement within 72 h of birth.

Exclusion criteria: 1. birth asphyxia (defined as umbilical artery / first hour arterial PH < 7.0 or cardiopulmonary resuscitation in the delivery room); 2. birth complicated by severe gastrointestinal malformations (such as intestinal atresia, esophago-tracheal fistula, intestinal rotation abnormalities, congenital megacolon); 3. prenatal diagnosis of congenital chromosomal abnormalities or suspected congenital genetic metabolic diseases; and 4. maternal drug abuse or contraindications to breastfeeding (HIV and cytomegalovirus infection).

### Collection of milk and oropharyngeal administration procedure

Obstetric nurses will encourage the mother of each newborn to start lactation within 24 h of delivery and educate them on breast massage and electric breast suction every 3 h. Mothers of preterm infants will be encouraged to milk frequently with hygienic hands to ensure a steady supply of breast milk. To ensure the feasibility of oropharyngeal administration of mother’s milk to preterm infants in the treatment group, the mother’s milk will be collected in a pre-labeled sterile milk collection bag. A minimum colostrum volume of 1.6 ml will be collected and stored in the breast milk refrigerator of NICU. After ascertaining that baby's information is consistent with details in the label on the breast milk storage bag, an on-site investigator (nurses preparing syringes) will select the storage bag with the latest lactation time and use sterile gloves and syringes to suck 0.1 ml of colostrum or sterile water for oropharyngeal administration, and cover them with opaque tape and needle cap. Each syringe will be labeled with serial number, hospitalization number, name and date. Then, the bag will be stored at 0 ~ 4℃ for 24 h for subsequent intervention. In this study, all other storage bags will be stored in a freezer at -18 °C until the infant begins enteral feeding.

In the first 48–72 h the treatment group will receive the treatment measures: two 1 mL sterile syringes each aspirated with 0.1 mL of colostrum, placed at room temperature for 5 min. The colostrum will be maintained at room temperature for OAC use. After removing the tip and cap of the first syringe, the colostrum will be slowly injected along the right oral mucosa for at least 20 s. Then, the right buccal mucosa will be wiped with a sterile cotton swab for at least 10 s. The second syringe will be used in the same way on the left oral mucosa. The procedure will be performed every three hours for five days. The control group will be treated with sterile water following similar operation steps. Colostrum for the control group will be frozen in the NICU breast milk refrigerator for enteral feeding. Vital signs will be monitored throughout the process, including heart rate, respiration, body temperature and pulse oxygen saturation. During treatment, newborns will be regularly monitored and observed. If their clinical symptoms are unstable (heart rate is greater than 200 or less than 100, breathing is greater than 80, or the oxygen saturation is maintained above 85% only when the oxygen concentration increases by 10%) [44], the intervention will be interrupted. Once the clinical situation stabilizes, the treatment will be resumed immediately, and the final intervention dose and adverse reactions recorded.

## Outcomes

Primary outcome measures: 1a) a between-group difference in gut microbial alpha diversity will be measured using Shannon diversity index at the 7th day and 1b) a between-group difference in the concentration of fecal metabolites (short chain fatty acids) will be quantitatively measured using non-targeted liquid chromatography-mass spectrometry (LC–MS) at the 7th day.

Secondary outcome measures: 2a) between-group differences in other diversity indicators of gut microbiota, including Simpson index (an indicator of species richness and evenness less affected by rare species than Shannon index) and Chao1 index (estimated total number of species) at the 7th day; 2b) between-group differences in alpha diversity of gut microbiota measured using Shannon index, Simpson index and Chao1 index on the 14th, 21st and 28th days of life; 2c) Between-group differences in the concentration of fecal metabolites quantitatively measured by non-targeted LC–MS at the 14th, 21st and 28th days of life; 2d) proportion of gut microbiota at phylum and genus level; 2e) gut microbial beta diversity explaining between-sample dissimilarity calculated using the Vegan package; and 2f) correlation between the comparative dominant flora and metabolites (Table 1).

**Table 1:** Outcomes, measures and methods of analysis

Outcome	Hypothesis	Outcome Measure	Methods of Analysis
**1.Primary** 1a A between-group difference in Shannon diversity index at the 7th day	OAC increases Shannon diversity and metabolites of gut microbiota	Shannon index (continuous)	Individuals: Wilcoxon text; Intergroup: T/Wilcoxon text
1b A between-group difference in fecal metabolites at the 7th day		Short chain fatty acids (continuous)	Individuals: FC, PCA/PLS-DA Intergroup: T/Wilcoxon text
2.** Secondary** 2a Between-group differences in other alpha diversity indexes (Simpson, Chao1) at the 7th day	OAC increases other alpha diversity and beta diversity, and changed the proportion of gut microbiota from birth to day 28. There is a Correlation between dominant microbiota and metabolites	Simpson index, Chao1 index (continuous)	Individuals: Wilcoxon text; Intergroup: T/Wilcoxon text
2b Between-group differences in Shannon index, Simpson index and Chao1 index at the 14th, 21st and 28th days of life		Shannon index, Simpson index, Chao1 index (continuous)	Individuals: Wilcoxon text; Intergroup: T/Wilcoxon text
2c Between-group differences in the concentration of fecal metabolites at the 14th, 21st and 28th days of life		Short chain fatty acids (continuous)	Individuals: FC, PCA/PLS-DA Intergroup: T/Wilcoxon text
2d Proportion of gut microbiota (phylum and genus level)		The percentile of gut microbiota at the phylum and genus levels of the total microbiota (binary)	Individuals: Wilcoxon text; Intergroup: Chi-square test
2e Gut microbial beta diversity		Bray–Curtis distances with 9999 permutations (continuous)	PCOA; LDA
2f Correlation between dominant microbiota and metabolites		Correlation between dominant microbiota and metabolites	Partial Least Squares Discriminant Analysis
**3.Subgroup Analysis**			
≤ 28w vs. > 28w	Gestational age affects adherence	All outcomes	Same as above

*OAC* oropharyngeal administration of colostrum, *FC* fold change, *PCA* principal component analysis, *PLS-DA* partial least squares discriminant analysis, *PCOA* principal co-ordinates analysis, *LDA* linear discriminant analysis

Subgroup analysis will be performed on preterm infants with gestational age ≤ 28 weeks (Table 1).

### Statistical analysis

Intention to treat (ITT) method will be used for statistical analysis. Baseline maternal and infant demographic and clinical characteristics of OAC group and placebo group will be compared using chi-square or Fisher’s exact test (such as infant sex, mode of delivery and presence or absence of chorioamnionitis) and t-test or Wilcoxon test for continuous variables. After removing low-quality sequences, original data will be analyzed following the steps of 16S rRNA discovery, clustering and identification. The number of operational taxonomic units (OTUs) will be calculated for each sample at a 97% sequence similarity level. A specific taxonomic unit will represent a specific species. Subsequently, a sparse curve will be drawn based on the results of OTUs generated using sample sequencing. Wilcoxon test will be used to analyze the diversity and richness of microbiota within individuals. A t-test or Wilcoxon test will be used to compare alpha diversity (Shannon index, Simpson index, and Chao1 index) between the two groups of infants with and without OAC intervention within four weeks of birth. Repeated measures analysis of variance (ANOVA) (for normally distributed data) or Kruskal Wallis test (for non-normal data) will be used to evaluate the microbial alpha diversity at different time points. The proportion difference of gut microbiota at the phylum and genus levels will be calculated using chi-square or Fisher's exact test. Values with *p* < 0.05 will be considered statistically significant. Beta diversity will be calculated using the Vegan package, and a principal co-ordinates analysis (PCOA) based on Bray–Curtis distances will be plotted. Adonis permutational multivariate analysis of variance of Bray–Curtis distances with 9999 permutations will be used to compare microbial community structure between each of the two groups.

Redundancy analysis (RDA) will be performed using the Vegan package to define the factors that could significantly affect gut microbial composition in each group. We will use Linear discriminant analysis (LDA) of effect size (LEfSe) to determine the most discriminant taxa between the two groups. If the effect of OAC intervention on gut microbiota is less than that of other confounding factors (delivery mode, antibiotic use, feeding type, etc.), we will adjust it with RDA.

Gut microbiota can regulate the signal pathways regulating intestinal mucosal homeostasis through the production of metabolites. Non-targeted LC–MS will be used to evaluate short chain fatty acids and other organic acids and alcohols. The relative and absolute concentrations of metabolites will be calculated using fold change (FC) value, and the difference in metabolite expression between the two groups will be explored. Principal component analysis or partial least squares discriminant analysis will be used to compare different metabolites among the sample types. A time dynamic curve will be used to further analyze metabolites of interest. The correlation between the relative abundance of dominant bacterial taxa based on 16S rRNA gene sequencing and the intensity of metabolites of interest will be determined using sparse partial least squares regression [45].

Details of all methods of analysis are provided in Table 1.

### Sample size

The purpose of this study is to detect the alpha diversity of gut microbiota in preterm infants (Shannon index is a better indicator of species richness and evenness) and group differences in short chain fatty acids of interest. At present, no study has reported the effect of OAC on the difference in alpha diversity of gut microbiota and metabolites between preterm infants receiving oropharyngeal administration of colostrum and those receiving sterile water. Therefore, it is difficult to estimate the expected change. However, clinically relevant differences in the Shannon index have been determined based on previous studies on the effect of different feeding types on the Shannon index of preterm infants at the 7th day of age [46–49]. According to these studies, the mean between-group difference in Shannon index ranged from 0.2 to 0.6 with a standard deviation of 0.2 to 0.8 and a mode of 0.6 [46–49]. In a study of the effect of OAC on oral microbiota, the mean between-group difference in Shannon index ranged from 0.2 to 0.5 [50]. Combined with these basic data, the mean difference in Shannon index is assumed to be 0.2, with a standard deviation of 0.6. We conducted sample size estimation using PASS 15.0 (NCSS, Kaysville, Utah, United States). To achieve a statistical power of 80% (2-sided type 1 error of 0.05), the calculated sample size of 143 patients for each group (286 in total) with a ratio of 1:1 will be used. In addition, the relevant difference in short chain fatty acids is based on the previous difference of acetic acid, the main metabolite of preterm infants at the 7th day of life with different feeding types. The mean value of the difference between groups of acetic acid is about 0.025 µmol/g, with a standard deviation of 0.02–0.1 for each group [51, 52]. Therefore, a sample of 92 infants in each group (184 in total) will provide 80% power to detect a difference in short chain fatty acids of at least 0.025, assuming a standard deviation of 0.06 and a 2-sided type I error of 0.05. In combination with these two sample sizes, we will use a relatively larger sample (143 cases). Assuming a loss of 10–12% to follow-up, we will need to include 160 infants in each group, resulting in a total sample size of 320. The sample size will be adjusted based on the results of interim analysis.

### Participant recruitment and informed consent

Data collection will begin in October 2022 and is expected to end in September 2023. Shenzhen People’s Hospital is the pilot research unit, and the pilot research will be carried out from August to September 2022. The annual census of very preterm infants in this facility is relatively high and the breastfeeding rate is very high (above 80%), hence ideal for the recruitment of subjects. Mothers who meet the inclusion criteria (fetal weight < 1500 g estimated by ultrasound) will be invited to participate. To achieve full registration of participants and target sample size, breast-feeding education will be provided in obstetrics to improve lactation awareness. Professional staff will guide pregnant women and provide them with breast care (such as breast massage). The principal investigator (PI) will explain the study in detail and respond to any questions raised. Mothers will be informed of their right to withdraw from the intervention at any time. They will also be informed that if they decline to participate in the study, their care and that of their infants will not be affected.

Written informed consent will be obtained from the patient's guardian. This study complies with the Declaration of Helsinki.

### Randomization

A 1:1 block randomization scheme will be adopted. Each group will be stratified by birth gestational age (< 28 weeks, 28 weeks, and 32 weeks). A random serial number will be randomly generated for each layer using SAS (v9.4 SAS Inst. Inc., Cary, NC) before the start of the study. The block length will be 6. Statisticians will prepare sealed, numbered, opaque envelopes for each site and hand them over to the study coordinator, who will ensure that the envelopes are used in a numerical order. After receiving the notice, the study coordinator will open the envelope, take down the group assignment form, copy the form and send it to the statistician. The manuscript will be left in the case file of the preterm infant. To achieve blinding, the study coordinator preparing the syringe (colostrum or placebo) will be different from the nurse conducting the intervention. The subjects, intervention nurses, the principal investigator and the data verifier will be blinded. During the pilot test, non-fixed codes will be used for blinding to represent each allocation scheme to prevent unblinding by inadvertently losing blindness to all trial participants.

### Data collection

Descriptive and clinical data be collected from both groups. Birth status characteristics, such as delivery mode, gestational age, weight and length, Apgar score will be collected in addition to the mothers’ occupation, education level, disease, use of drugs, supplements and antibiotics. During hospitalization, clinical data will be collected on enteral feeding (start time, duration, starting dose, feeding type and total enteral feeding time); parenteral nutrition (start time and duration); antibiotics use in infants (type, start time, duration and dose); NEC, LOS and mortality; and respiratory support.

We hypothesize that OAC would regulate gut microbiota and its metabolites in very preterm infants, resulting in an increase in microbial diversity, with increasing dominance of Lactobacillus/Bifidobacterium predominating and a relative decrease in Proteus species. Stool samples will be collected from selected subjects at the first defecation, as well as on the 7th, 14th, 21st and 28th days and immediately frozen at − 80 °C. Fecal samples refrigerated with dry ice will be transported to the Microbiology Research Institute of Shenzhen People’s hospital. After extracting DNA from frozen fecal samples, 16S rRNA gene will be amplified by polymerase chain reaction (PCR) (v3-v4 hypervariable region) and sequenced using Illumina miseq platform. All data collection procedures will be performed following the local coronavirus disease 2019 (COVID-19) epidemic prevention and control policy.

### Missing data

Retention of participants will be ensured during the monitoring period through the support of mothers, lactation encouragement and provision of psychological services. The following events are considered as follow-up losses: the study is not continued due to neonatal death, unstable clinical symptoms and other reasons; the intervention dose does not reach the expected 70%; and parents of preterm infants withdraws from the trial at any time. Eliminated or corrected observations and related reasons will be recorded for future reference.

Most types of missing data in the study will come from random missing. All missing data will be processed with SAS software using the double-robust inverse probability weighting method [53]. Its underlying principle is to estimate the weighted average of the outcome indicators of the complete data sample. Among them, the weight of each individual is the reciprocal of the probability that the outcome index of the individual is not missing, yielding an effect estimation closer to the real value.

### Data management

The purpose of data management is to ensure the reliability, integrity and accuracy of data. All study participants will be given a study identification number. Data from each research center will be imported to the collected electronic data capture (EDC) system and protected by password. These data will be verified, corrected, blind audited and locked. The backup is scheduled at midnight every day. Shenzhen People’s Hospital, as the supervision unit, will provide a detailed definition of each variable in the EDC system and ensure consistent data entry. The research team's access to participant information will be strictly limited to the purpose of running the study. In future studies, the storage and use of these data will require additional consent of both parents. For this analysis, the researchers will have access to the finalized test data set.

### Quality control

**Fig. 2:**
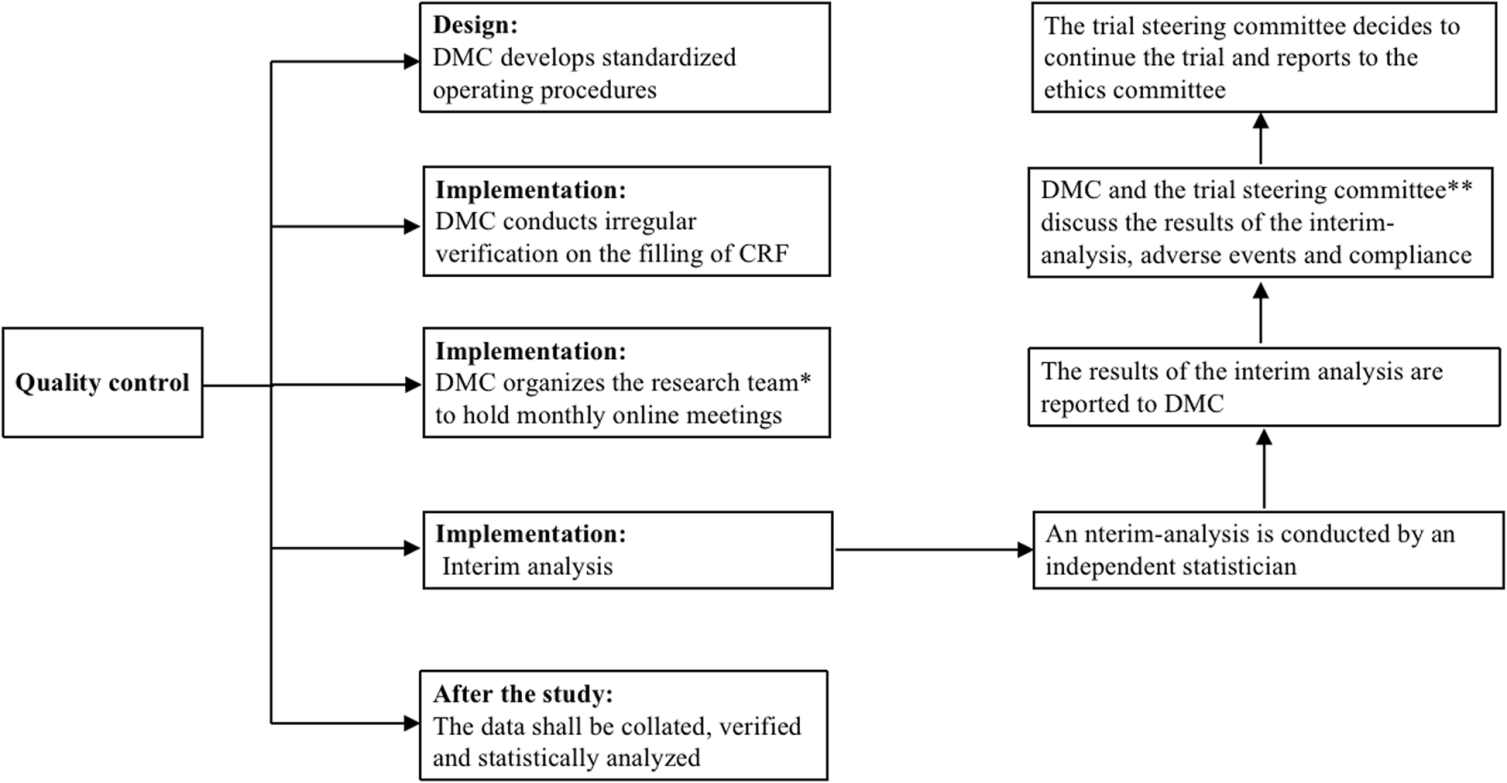
Quality control process of the protocol. DMC, data monitoring committee (composed of data verifiers at the coordination center and the principal investigators at each center); CRF, case report form. * The research team of each center is composed of the principal investigator, data quality controller, syringe preparing nurse and the intervener. ** The trial steering committee (composed of the directors of neonatal pediatrics at each center and the heads of the medical safety review team)

In clinical research, quality control should be carried out in the whole process of trial design, implementation and completion. In the design stage, to improve the quality of test data, a data monitoring committee (DMC) will be established. DMC will composed of the data verifiers (ZY and NW) and the principal investigators at each center (XY, RL and HP). The research team at each center will be composed of the principal investigator (XY, RL and HP), data quality controller (CC, LZ and ZY), syringe preparing nurse and the intervener. In the design phase, calculation of sample size, training of researchers, formulation of data management plan, and reasonable selection of efficacy observation indicators will be considered. The training will cover standardized operation process, including recruitment, randomization and web-based data collection. In the implementation phase, a standardized data collection process will be adopted, and the DMC will conduct regular verification of the filling of case report form (CRF) and research records at each center. Data verification will mainly focus on the following aspects: 1) the timeliness and completeness of data filling in CRFs, and the education on mother lactation to minimize data loss; 2) normalization of data filling; 3) records of adverse events, including the number and duration of adverse events during OAC, and records of colostrum intervention; and 4) monitor the progress of the study and verify the implementation of the study protocol, blind method performance and adverse events. After the study, the data will be collated, verified and statistically analyzed (including missing values, inclusion and exclusion criteria, and adverse events). The quality control process of the protocol is shown in Fig. 2.

The whole research team will hold online meetings every month through multimedia video conferencing software to discuss the implementation of the scheme as needed and quickly solve any problems. When 50% of randomly assigned patients completed five stool samples collection, the primary endpoint was analyzed in the interim. An interim-analysis will be conducted by independent statisticians without knowledge of treatment allocation. Statisticians will report to the DMC. The DMC will conduct non-blind access to all data and discuss the results of the interim analysis, adverse events and compliance with the trial steering committee (composed of the directors of neonatal pediatrics at each center and the heads of the medical safety review team) at the multicenter meeting. Adverse events are defined as aspiration to the lungs, resuscitation due to severe bradycardia or apnea after intervention, and postnatal infection with cytomegalovirus thought to be associated with fresh breast milk. If cases related to these adverse events occur, we will intervene immediately. To ensure the safety of the included infants, vital signs will be carefully monitored during oropharyngeal administration. If the vital signs are unstable, the intervention will be stopped in time. The trial steering committee will decide to continue the trial and report to the ethics committee, which is independent of the sponsor and investigators (Fig. 2).

### Ethical and dissemination

Our protocol has been approved by the medical ethics committee of each hospital participating in the recruitment. Ethical approval will be provided before each center starts recruiting. Informed consent, participant education, recruitment materials and subsequent modifications will also be reviewed and approved by the ethics committee. Researchers trained in this method will explain the research objectives, risks and postnatal benefits to the mothers. The guardians of all subjects will be provided with educational materials and will sign the informed consent form. The researcher will submit the safety and progress report to the ethics committee at least every year and three months after the termination or completion of the research, as well as data on the safety and effectiveness review by the data monitoring committee. The results of this study will be published in academic conferences and peer-reviewed open access journals.

## Discussion

This multicenter, double-blind RCT study will be performed based on 16sRNA gene sequencing and non-targeted metabolic mass spectrometry. The purpose of the trial is to evaluate the effect of OAC on the gut microbiota and metabolites of preterm infants.

OAC often occurs before or in the early stage of enteral feeding of preterm infants. Previous studies have confirmed that OAC is safe and effective [54]. It increases IgA, IgM, and lactoferrin, to regulate the immune function [55]. The role of human microbiota and metabolic activities in health and disease have been revealed through the application of new technologies. However, the effect of OAC on gut microbiota and metabolites is not clear. Previous studies have suggested that microbiota regulate the development of the immune system and brain in newborns [56]. Therefore, we hypothesized that OAC can increase intestinal bacterial diversity, promote the abundance of Bifidobacteria and other dominant microbiota, as well as the production of short chain fatty acids, thereby reduce vascular damage caused by oxidative stress [57]. It can also protect the intestinal barrier integrity and prevent the occurrence of preterm infant-related diseases by modulating the brain-gut axis.

It should be noted that the gut microbiota are affected by many factors. Previous studies have shown that infants receiving antibiotic treatment have increased relative abundance of Proteobacteria, and decreased the relative abundance of Actinobacteria, Firmicutes, and Bacteroidetes decreases [23]. The relative abundance of Bacteroides decreased after cesarean section and was higher during vaginal delivery [58]. Formula-fed infants showed higher relative abundance of Firmicutes, whereas breast-fed infants showed increased abundance of the phyla Actinobacteria and the genus Bifidobacterium [59]. Therefore, we plan to conduct a multicenter, double-blind RCT study to evaluate the effect of OAC on the gut microbiota and metabolites of preterm infants. The design of the proposed study considers group differences in mode of delivery, antibiotic exposure, feeding type, among other factors to evaluate the effectiveness of OAC comprehensively. The relationship between bacterial species composition and environmental variables is determined by controlling some environmental factors which affect gut microbiota imbalance using statistical methods such as RDA.

Lactational stages are categorized as colostrum (≤ 5 days postpartum), transitional milk (6–15 days postpartum), and mature milk (≥ 16 days postpartum) [3, 60, 61]. In addition, the peak of IgA in human milk has been reported to range between the fourth and fifth days after delivery [62]. Therefore, the duration of OAC is set to 5 days in the proposed study. However, the OAC program is not unified in previous studies. Therefore, whether a longer duration results in optimal intestinal microbiota regulation deserves further discussion. Another limitation is that we intend to use fecal samples for non-targeted LC–MS analysis. As fecal substances are such a complex matrix, the discovery of biomarkers is more susceptible to inter individual variation (metabolites and confounding factors) and differences in analytical methods [63]. Although metabolome analysis of blood, urine, saliva, and other tissues combined with nuclear magnetic resonance (NMR), gas chromatography-mass spectrometry, and other analytical techniques will yield valuable results [31], these techniques are expensive and complex.

The study population will be recruited from three large NICU. This study advocates administration of early colostrum to preterm infants and encourages breast-feeding to improve the health outcomes of preterm infants because early colostrum enriches gut microflora.

### Trial status

This is a current, ongoing trial which is actively recruiting study participants. We expect to finish patient recruitment in September 2023 and will present the final results during 2024.

## Data Availability

Not applicable.

## Abbreviations

OAC: Oropharyngeal administration of colostrum
NICU: Neonatal intensive care unit
LC–MS: Liquid chromatography-mass spectrometry
NEC: Necrotizing enterocolitis
LOS: Late onset sepsis
ITT: Intention to treat
OTU: Operational taxonomic unit
ANOVA: Analysis of variance
PCOA: Principal co-ordinates analysis
LDA: Linear discriminant analysis
RDA: Redundancy analysis
FC: Fold change
PCA: Principal component analysis
PLS-DA: Sparse partial least squares
SPLs: Partial least squares-discriminant analysis
PI: Principal investigator
PCR: Polymerase chain reaction
COVID-19: Coronavirus disease 2019
EDC: Electronic data capture system
CRF: Case report form
DMC: Data monitoring committee
RCT: Randomized controlled trial
NMR: Nuclear magnetic resonance
